# SUL-138 mitigates accelerated endothelial aging and protects the kidney

**DOI:** 10.1042/CS20255735

**Published:** 2025-11-06

**Authors:** Annika A. Jüttner, Sabrina Ribeiro Gonsalez, Martijn H. van Heugten, Ehsan Ataei Ataabadi, Keivan Golshiri, Ewout J. Hoorn, Marian Clahsen - van Groningen, Rene de Vries, Ingrid M. Garrelds, Dennis Schutter, A. H. Jan Danser, Adrianus C. van der Graaf, Daniël H. Swart, Leo E. Deelman, Robert H. Henning, Jenny A. Visser, Guido Krenning, Anton J.M. Roks

**Affiliations:** 1 Division of Vascular Medicine and Pharmacology, Department of Internal Medicine, Erasmus MC, University Medical Center, Rotterdam, Netherlands; 2 Division of Nephrology and Transplantation, Department of Internal Medicine, Erasmus MC, University Medical Center, Rotterdam, Netherlands; 3 Department of Pathology, Erasmus MC, University Medical Center, Rotterdam, Netherlands; 4 Sulfateq B.V., Groningen, Netherlands; 5 Department of Clinical Pharmacy and Pharmacology, University Medical Center Groningen, University of Groningen, Groningen, Netherlands; 6 Division of Endocrinology, Department of Internal Medicine, Erasmus MC, University Medical Center, Rotterdam, Netherlands

**Keywords:** aging, endothelial cells, mitochondria, SUL-138, tubular injury, vasodilation

## Abstract

Vascular aging is marked by increased levels of reactive oxygen species in endothelial cells. Reactive oxygen species can, among others, be produced by dysfunctional mitochondria, contributing to acceleration of vascular aging by promoting DNA damage response and senescence. In the aged vasculature, impaired endothelial cell function causes decreased vasodilation, which may also have an impact on peripheral organs such as the kidney. The aim of this study was to investigate the effect of chronic treatment with SUL-138 (30 mg/kg/day), a novel mitochondrial protective compound, on DNA damage-prompted, accelerated endothelial aging and associated kidney dysfunction in mice. Endothelial-specific aging was induced by knock-out (KO) of DNA repair endonuclease Ercc1 in mice [endothelial-cell specific Ercc1 KO (EC-KO) mice]. We showed that impaired endothelium-dependent vasodilation and expression of DNA damage response markers in EC-KO mice were restored after the treatment with SUL-138. The underlying mechanism of improved vasodilation was an increase in endothelium-derived hyperpolarization (EDH). Endothelial-specific aging induced tubular injury, sodium wasting, and increased inflammatory markers in the kidney, which were normalized by the treatment with SUL-138. We conclude that accelerated endothelial aging adversely affects vascular function and causes kidney tubular injury. SUL-138 rescues endothelial aging, restores vasodilation by increasing EDH, and protects the kidney. Thus, preservation of mitochondrial function is a potential pharmacotherapeutic target in aging-related dysfunction provoked by the DNA damage response.

## Introduction

Aging is a major risk factor for developing cardiovascular and kidney diseases, which are leading causes of mortality and morbidity in developed countries [1–3]. Aging of the vascular system is an inevitable process that can take place independently from the impact of cardiovascular risk factors like obesity, diabetes, and smoking [1,4,5]. Moreover, non-occlusive vascular aging is assumed to be a major driver of age-related diseases such as chronic kidney diseases [6]. Local renal endothelial cells (EC) are important regulators for renal blood flow and oxygen and nutrient supply, which play a pivotal role in maintaining proper glomerular filtration and tubular salt reabsorption [3,7–9]. Consequently, EC dysfunction contributes, among others, to the pathophysiology of chronic kidney diseases [10]. Pharmacological targeting of vascular aging in general and endothelial aging in particular could therefore be used as a primary prevention strategy for age-related kidney diseases. A potential target of anti-aging therapies includes the modulation of bioenergetic processes, in which the mitochondria play a key role [11]. Dysfunction of mitochondria, in particular production of excessive amounts of reactive oxygen species due to elevated mitochondrial electron leakage [11], is an important hallmark of aging [12]. Whether this is cause, consequence, or both at once in the biology of aging is an ongoing question [12], but also a possible fundament to design therapies against vascular aging and its resulting kidney disorders.

Exacerbated mitochondrial electron leakage in the aged vasculature directly interferes with vascular signaling as it decreases nitric oxide (NO) bioavailability in EC, leading to vascular dysfunction [1,13,14]. NO is predominantly known for its pivotal importance in vasodilation, although it has also been implicated in vascular permeability, vascular smooth muscle cell quiescence, as well as anti-thrombotic and anti-inflammatory effects [15]. Related to vasodilation, the loss of NO can be compensated by increased endothelium-derived hyperpolarization (EDH) partially rescuing vasodilation [16–18]. EDH acts through hyperpolarization of the cell membrane through opening of small and intermediate Ca^2+^-dependent potassium channels [small Ca^2+^-dependent potassium (SK_Ca_)/intermediate Ca^2+^-dependent potassium (IK_Ca_)] [19]. Interestingly, during aging, the regulation of these channels at least partly depends on mitochondrial Ca^2+^ [20]. Pharmacological modulation of mitochondrial function could therefore preserve both NO and EDH signaling and thereby support vascular function during aging.

Alternatively, to a direct hindering of vasodilation, excessive mitochondrial electron leakage has been implicated in DNA damage. DNA damage accumulation and the resulting DNA response that is marked by cellular senescence is a causative factor in vascular aging [12,13,21,22]. Accumulated DNA damage causes widespread metabolic and inflammatory responses, as well as a switch to cellular repair and senescence [12,23,24]. Additionally, inflammatory cytokines such as TNF-α, which are part of the DNA-damage response, can reduce mitochondrial complex I activity, which in turn leads to increased mitochondrial superoxide production, resulting in a vicious cycle [12,25]. Therefore, modulation of mitochondrial function could preserve vascular function by targeting DNA damage and the resulting response.

SUL compounds are modified 6-chromanols that preserve mitochondrial function by activating mitochondrial complex IV [26] and thereby maintain mitochondrial ATP production as well as reduce mitochondrial superoxide production [26–28]. SUL compounds thereby protect against kidney and vascular injury as well as conditions of metabolic stress in various pre-clinical models [27–30]. Given the causal role of mitochondrial dysfunction in aging in general and vascular aging in particular, we hypothesize that SUL-138 prevents vascular aging due to protection against the DNA damage response, and thus protects against kidney dysfunction.

To test our hypothesis, we used an established accelerated aging mouse model yielded by knock-out (KO) of DNA repair endonuclease excision repair cross-complementing rodent repair deficiency, complementation group 1 (Ercc1), which leads to inhibition of DNA repair and thereby DNA damage accumulation and the detrimental response that causes aging [31–33]. To more specifically study vascular aging, we developed a mouse model where Ercc1 was exclusively knocked out in EC (EC-KO mice) [13]. EC-KO mice display an endothelium-specific DNA damage response, which within five months leads to widespread endothelial dysfunction and vascular aging markers, observed in aorta, iliac and coronary artery, and in the microvasculature, among which are peritubular microvascular leakage and papilla damage in the kidney [13]. Thus, EC-KO mice are a model to test drug effects on vascular aging and concomitant renal dysfunction in a reasonable time frame.

In this study, we investigated the effect of SUL-138, a novel protective drug that preserves mitochondrial function, on DNA damage-related endothelial aging in EC-KO mice, with a focus on vascular and kidney function. Effects on NO and EDH were measured as potential mediating mechanisms of vascular improvement.

## Material and methods

### Animal model

Mice were bred as described previously [13]. In brief, the Cre-loxP system was used to generate a conditional mouse model to knockout endonuclease *Ercc1* in EC (B6.Cg-Tg(Tek-cre)12Flv/J, The Jackson Laboratory). In the resulting litters, Tie2cre^+^ Ercc1^fl/−^ mice have an in EC and are referred to as EC-KO mice throughout this manuscript. Tie2cre^+^ Ercc1^fl/+^ mice were used as wildtype (WT) littermate controls.

Male and female mice (50:50 per group) were housed in groups in ventilated cages with 12 h light/dark cycles with water and food *ad libitum*. Oral treatment (30 mg/kg body weight/day) [30] with SUL-138 (6-hydroxy-2,5,7,8-tetramethylchroman-2-yl)(piperazin-1-yl)methanone; provided by Sulfateq B.V., The Netherlands) started at the age of 14 weeks, when vascular dysfunction started to develop in EC-KO mice [13], until euthanasia at the age of 22 weeks. A total of 10 mmol/l SUL-138, dissolved in ethanol and diluted in water (8 EC-KO vs. 7 WT SUL-138 treated) or 0.0015% (v/v) ethanol for the vehicle-treated mice (12 EC-KO vs. 12 WT), was sprayed homogeneously over the food pellets and dried overnight (as described previously [34]). SUL-138 plasma levels were measured from blood drawn at euthanasia and were on average 197 ± 39 µg/l, similar to a previous study [34]. SUL-138 plasma levels in vehicle-treated mice were non-detectable.

As previously validated [13], successful EC-KO was confirmed in lung tissue where the ERCC1 expression was significantly lower in EC-KO control (56 ± 7% of the protein level in WT, *P*<0.05) and EC-KO SUL-138 treated mice (61 ± 7% protein of WT) (Supplementary Figure 1A and Supplementary Figure 1D).

### Blood pressure

Blood pressure was measured at 20 weeks of age *in vivo* in conscious mice using a non-invasive tail cuff method, as described previously [13].

#### *Ex vivo* vascular function

Mice were euthanized by exsanguination under 3% isoflurane at the age of 22 weeks, and the thoracic aorta was collected in cold Krebs–Henseleit buffer (118 mmol/l NaCl, 4.7 mmol/l KCl, 1.2 mmol/l MgSO_4_, 1.2 mmol/l KH_2_PO_4_, 2.5 mmol/l CaCl_2_, 11 mmol/l glucose, 25 mmol/l NaHCO_3_, pH 7.4). Aortic segments were mounted in wire myograph setups (Danish Myo Technology (DMT)) (at 37°C and aerated with 95% CO_2_, 5% O_2_) and normalized as described previously [13]. Pathway inhibitors were added to determine NO-[l-NAME (10^−4^ mol/l) (N5751, Sigma-Aldrich)] and EDH contribution [apamin (10^−7^ mol/l) (A9459, Sigma-Aldrich) and TRAM-34 (10^−5^ mol/l) (T6700, Sigma-Aldrich)] to endothelium-dependent vasodilation. The acute effect of SUL-138 was assessed by pre-incubating with 100 µmol/l SUL-138 (Sulfateq B.V.) followed by pre-constriction with thromboxane A2 analogue U46619 (10^−8^ –10^−7^ mol/l) (Sigma-Aldrich). A parallel segment without inhibitors was pre-constricted and used as a control condition. Once a plateau was reached, acetylcholine (Sigma-Aldrich) was added cumulatively (10^−9^ –10^−5^ mol/l). Endothelium-independent vasodilation was evaluated by adding sodium nitroprusside (Merck) cumulatively (10^−11^–10^−4^ mol/l). Vasorelaxation was expressed in % relative to U46619 pre-constriction. Overnight SUL-138 incubation (30 µmol/l) was performed at 37°C while aerated with 95% CO_2_, 5% O_2_. On the next day, aortic segments were mounted in DMT wire myograph setups as described above.

### Metabolic cage experiments

At the age of 21 weeks, mice were put in metabolic cages with food and water *ad libitum*. After acclimatizing for ~9 h, the water bottles were weighed and the collection phase started. After 24 h, water bottles were weighed again, urine was collected, and mice were put back into their original cages.

### Urine and blood plasma analysis

Creatinine was determined by the QuantiChrom^TM^ Creatinine Assay Kit (DICT-500, BioAssay Systems), albumin in an ELISA (ab108792, Abcam), plasma copeptin was measured with Mouse Copeptin EIA (P01185, RayBio®), and urinary kidney injury marker 1 (KIM-1) was determined with the Mouse TIM-1/KIM-1/HAVCR Quantikine ELISA Kit (MKM100, R&D Systems), according to the manufacturer’s instructions. Creatinine clearance was calculated using the following formula: clearance=(urinary creatinine_24h_ × urine volume_24h_)/(plasma creatinine × 24 h). Electrolyte measurements were performed by the Clinical Chemistry Department (Erasmus MC, Rotterdam) on a Cobas 8000 modular analyzer (Roche Diagnostics). Plasma SUL-138 levels were measured by Sulfateq B.V. using LC-MS analysis.

### Protein expression

Proteins were isolated in homogenization buffer [0.3 mol/l sucrose, 50 mmol/l Tris-HCl pH 7.5, 1 mmol/l EDTA, 1 mmol/l EGTA, 1 mmol/l sodium-orthovanadate, 50 mmol/l sodium fluoride, 1 mmol/l DTT, 1 mmol/l Phenylmethylsulfonyl fluoride, 1% (v/v) Triton x-100, phosphatase inhibitor cocktail 3 (P0044, Sigma-Aldrich), and cOmplete^TM^ Protease Inhibitor Cocktail (11697498001, Roche)] and samples were normalized to the lowest protein concentration with 1× Laemmli buffer and incubated for 10 min at 65°C. In total, 35 µg (abdominal aorta) or 40 µg protein (kidney) and protein ladder (26619, Thermo Scientific) were then loaded on a gel, and when the required separation was reached, they were transferred to membranes (Trans-Blot Turbo 0.2 µm PVDF Transfer Pack, Bio-Rad) with the Trans-Blot Turbo Transfer System (Bio-Rad). Membranes were blocked with 5% (w/v) BSA or non-fatty milk for 2 h at room temperature (RT), followed by overnight incubation in primary antibody (listed in Supplementary Table S1) at 4°C. The membranes were then incubated for 1 h at RT in the secondary antibody [goat anti-mouse- or goat anti-rabbit-HRP conjugate (Bio-Rad, 1:3000)], and visualized with Clarity Western ECL substrate (Bio-Rad) following manufacturer’s instructions using an Amersham Al600.

## Phenylmethylsulfonyl Fluoride

, 1% (v/v) Triton x-100, phosphatase inhibitor cocktail 3 (P0044, Sigma-Aldrich), and cOmplete^TM^ Protease Inhibitor Cocktail (11697498001, Roche)] and samples were normalized to the lowest protein concentration with 1× Laemmli buffer and incubated for 10 min at 65°C. In total, 35 µg (abdominal aorta) or 40 µg protein (kidney) and protein ladder (26619, Thermo Scientific) were then loaded on a gel, and when the required separation was reached, they were transferred to membranes (Trans-Blot Turbo 0.2 µm PVDF Transfer Pack, Bio-Rad) with the Trans-Blot Turbo Transfer System (Bio-Rad). Membranes were blocked with 5% (w/v) BSA or non-fatty milk for 2 h at room temperature (RT), followed by overnight incubation in primary antibody (listed in Supplementary Table S1) at 4°C. The membranes were then incubated for 1 h at RT in the secondary antibody [goat anti-mouse- or goat anti-rabbit-HRP conjugate (Bio-Rad, 1:3000)], and visualized with Clarity Western ECL substrate (Bio-Rad) following manufacturer’s instructions using an Amersham Al600.

The Western blot technique is a semi-quantitative method where all analyzed protein samples should be put on the same gel. The variation between the EC-KO vehicle-treated mice requires a high sample size [10–12] to reach statistical significance to evaluate the effect of the genotype. Only the protein expression of WT and EC-KO vehicle-treated as well as EC-KO SUL-138-treated mice fits on one gel. We have omitted WT SUL-138-treated mice because this was the least relevant group.

### mRNA expression

RNA was isolated with the TRIzol method (Fisher Scientific) and used for the cDNA synthesis (Maxima H Minus First Strand cDNA synthesis, K1652, ThermoFisher) according to the manufacturer’s instructions. qPCR was performed with the CFX Opus Real-Time PCR System (Bio-Rad) using the SYBR^TM^ Green PCR Master Mix (4309155, Applied Biosystems^TM^) with *Gapdh* and *β-Actin* (mouse samples) or *Hprt1, Gapdh,* and *β-Actin* [human umbilical cord endothelial cells (HUVEC)] as housekeeping genes, measured as duplicates per sample (1× PCR Master Mix, 200 nmol/l forward primer, 200 nmol/l reverse primer, 10 ng cDNA). Used primers (self-designed and ordered fromIntegrated DNA Technologies (IDT)) are listed in Supplementary Table S2. Before using the primers to evaluate target mRNA expression, the primer PCR efficiency was tested using a cDNA dilution series (1:2 dilution series from 20 to 0.625 ng cDNA/well) in duplicates. To determine the PCR efficiency E, the average Ct per cDNA concentration was plotted over the corresponding log (cDNA concentrations) to calculate the slope of the curve. The individual PCR efficiencies per primer pair were calculated using the following formula: E=power(10; −1/slope). Relative quantity of the gene of interest was calculated using the 2^ΔΔCt^ method, adjusted for the calculated PCR efficiency of the individual primers.

### Histology and immunohistochemistry

The kidneys were processed for histology as described previously [13]. In brief, the tissue was first fixated in 4% [v/v] formaldehyde, cut transversally, embedded in paraffin, and sectioned at 3 µm. Kidneys were then stained with periodic acid–Schiff’s (PAS) staining for evaluation of glomerular parameters as described previously [29]. The tubular injury was scored by an experienced nephropathologist (M.C.C.v.G.) for acute tubular damage (10 magnification), using a five-point scale, according to the following criteria: tubular dilatation, cast deposition, brush border loss, and necrosis. Tubular damage was graded in ten fields with a score of 0–5: 0, no changes; 1, mild, <10%; 2, moderate, 10–25%; 3, severe, 25–50%; 4, very severe, 50–75%; and 5, extensive damage, >75%.

Immunohistochemical analysis was performed using the immunoperoxidase technique [avidin-biotinylated horseradish peroxidase H complex (ABC Elite Vectastain; Vector Laboratories) and 0.1% (v/v) 3,3′-diaminobenzidine tetrahydrochloride (Vector Laboratories)] as described previously [13] with p21 primary antibody (ab107099; 1:1000; Abcam) and biotin-conjugated goat anti-rat secondary antibody. From each mouse kidney, sections were analyzed using the NanoZoomer 2.0 HT device. An average of 20 microphotographs using the 20× objective was captured and quantified by ImageJ plugin 3.3.2, and the average of these values was expressed as arbitrary units ± standard deviation (SD).

### Cell culture experiments

HUVECs were kindly provided by the Precision Medicine in Oncology group (Erasmus MC, Rotterdam, The Netherlands) and cultured in Endothelial Cell Growth Medium (PromoCell) supplemented with the Endothelial Cell Growth Medium SupplementPack (PromoCell) (2% [v/v] fetal calf serum, 0.4% [v/v] endothelial cell growth supplement, 0.1 ng/ml human recombinant epidermal growth factor, 1 ng/ml recombinant human basic fibroblast growth factor, 90 µg/ml heparin, 1 µg/ml hydrocortisone). Senescence and mitochondrial dysfunction were induced by incubating for 24 h with 0.5 µmol/l doxorubicin [35–37] in the presence or absence of 1 µmol/l of the hydrochloride salt of SUL-138, followed by six days in cell medium without doxorubicin in the presence or absence of the hydrochloride salt of SUL-138.

### Statistics

All results are expressed as mean ± SD except for wire myography results, which are presented as mean ± standard error (SEM), and mRNA expression results which are presented as geometric mean ± geometric SD. Dose-response curves were analyzed with the general linear model (GLM) with repeated measures (IBM® SPSS® Statistics). Results with normal distribution (tested with Kolmogorov–Smirnov test) were analyzed using two-way ANOVA with post-hoc Bonferroni’s multiple comparisons test. Samples without normal distribution were analyzed with the Kruskal–Wallis test including multiple comparisons as post-hoc test. Western blot data were analyzed with ordinary one-way ANOVA with post-hoc Tukey’s multiple comparisons test or Kruskal–Wallis test including post-hoc multiple comparisons when not normally distributed. The tubular injury score was evaluated with a Mann–Whitney test (GraphPad Prism, Version 8.0.1).

## Results

### Vascular function

**Figure 1:**
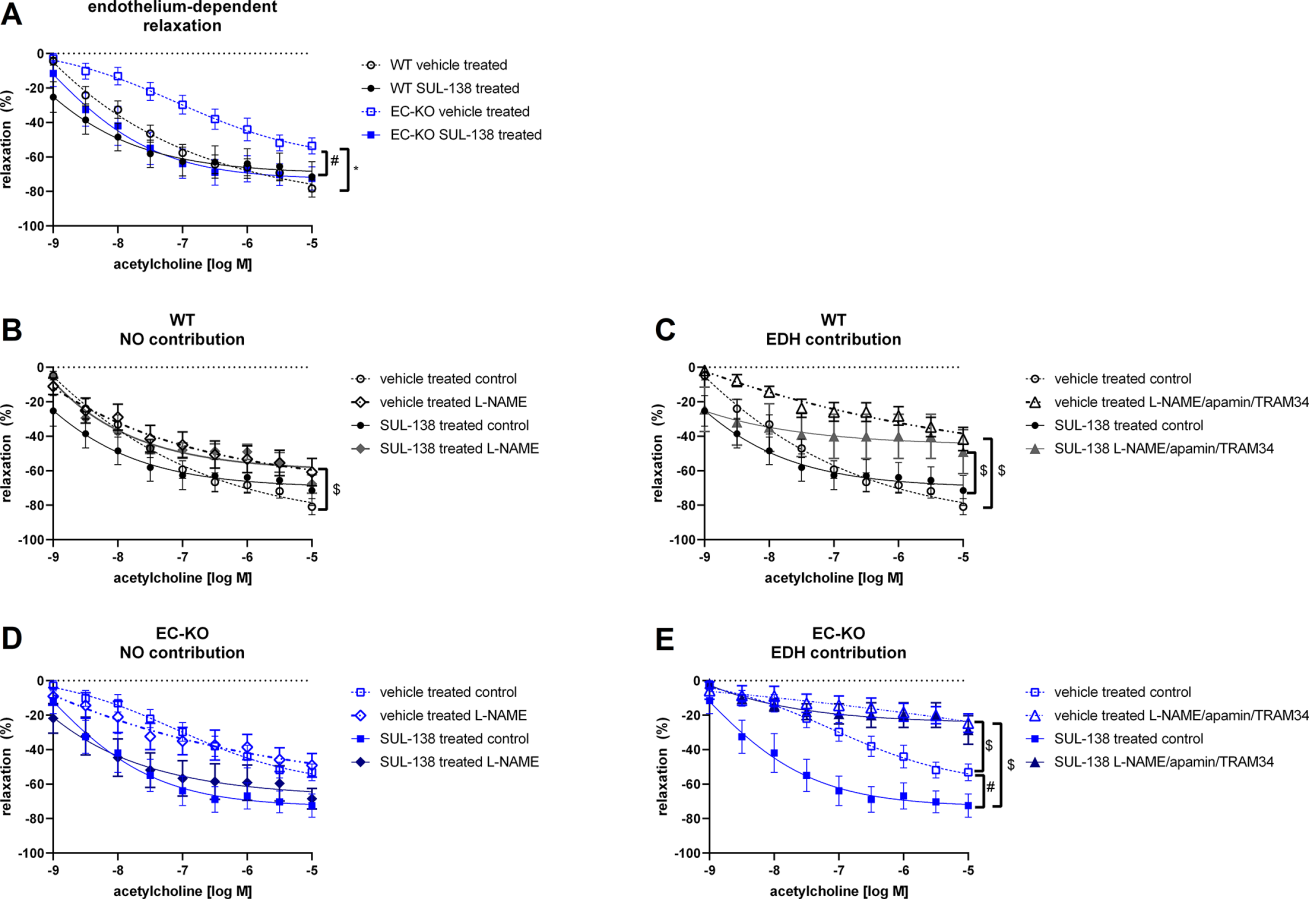
*Ex vivo* vascular function of thoracic aorta from endothelial cell-specific Ercc1 KO mice (EC-KO) and wildtype (WT) mice Endothelium-dependent vasodilation (**A**), pathway contribution in WT (**B and C**)and pathway contribution in EC-KO mice (**D and E**). ‘Control’ indicates measurement without inhibitor pre-incubation, ‘l-NAME’ indicates measurements in the presence of l-NAME, an inhibitor of endothelial nitric oxide synthase, and ‘l-NAME/apamin/TRAM-34’ indicates measurements in the presence of l-NAME and inhibitors of small and intermediate calcium-activated potassium channels (apamin, TRAM-34). Data are shown as means ± SEM. Significant effect of genotype: * or SUL-138: # or inhibitor: $ (GLM with repeated measures with *P*<0.05). WT vehicle treated *N*=11–12, WT SUL-138 treated *N*=5–6; EC-KO vehicle treated *N*=8–10; EC-KO SUL-138 treated *N*=5–6. EDH, endothelium-derived hyperpolarization; NO, nitric oxide.

Endothelium-dependent vasodilation was significantly decreased in EC-KO vehicle-treated mice compared with WT vehicle-treated mice, which was completely restored after chronic treatment with SUL-138 (Figure 1A). In WT vehicle-treated mice, acetylcholine-induced relaxation was partly composed of NO and partly mediated by EDH, which was not altered by the treatment with SUL-138 (Figure 1B and C). In EC-KO vehicle-treated mice, EDH was the major contributor to vasodilation (Figure 1D and E). SUL-138 treatment did not affect the lowered NO contribution in EC-KO mice (Figure 1D), yet substantially increased EDH contribution (Figure 1E). EDH-mediated vasodilation involved activation of SK_Ca_ and IK_Ca_ channels in the EC-KO mice, as shown by their respective inhibition (Figure 1E).

**Figure 2:**
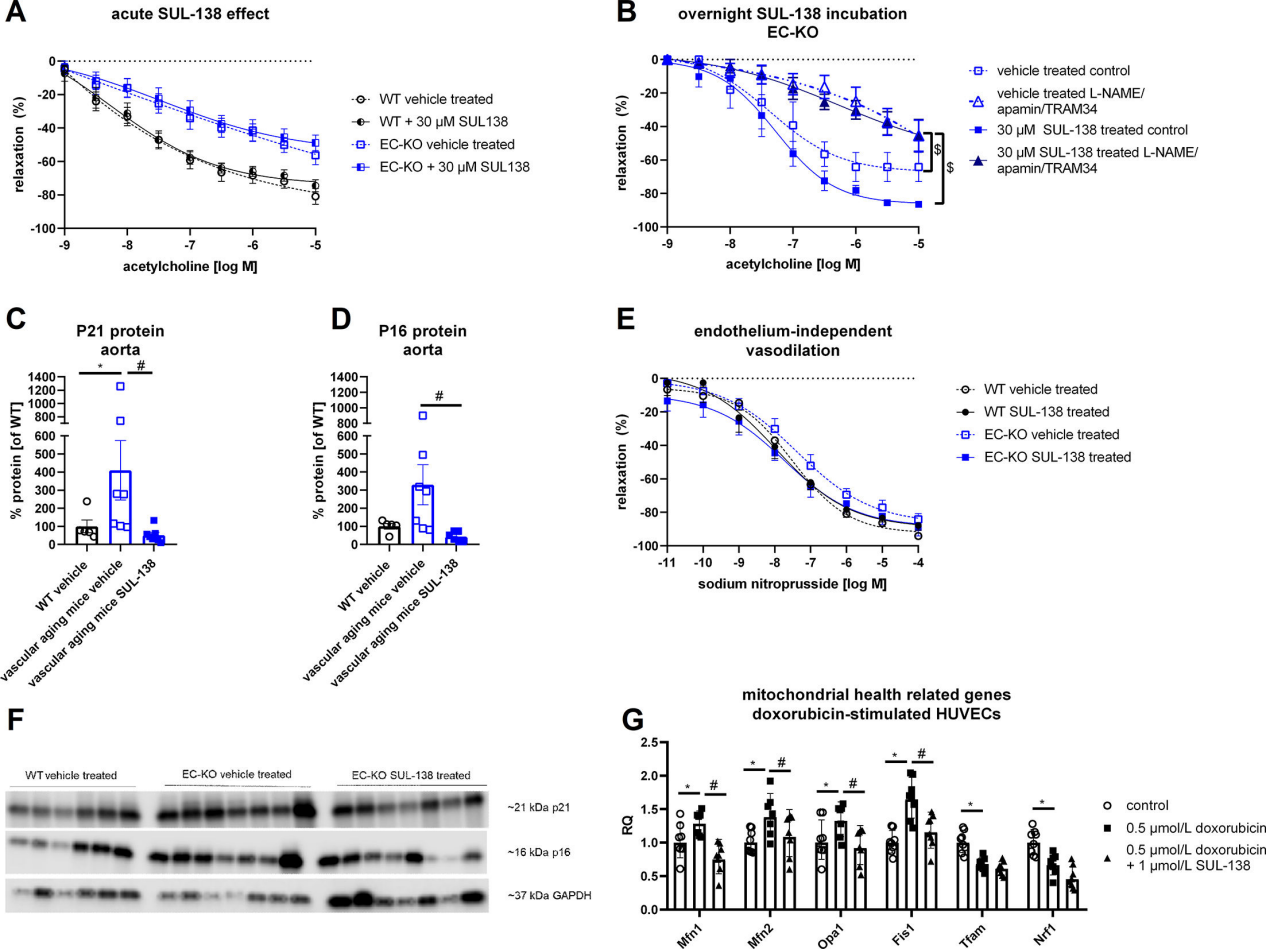
*Ex vivo* vascular function of thoracic aorta from endothelial cell-specific Ercc1 KO mice (EC-KO) and wildtype (WT) mice and senescence markers in aorta Effect of acute incubation with SUL-138 (**A**) and overnight incubation with SUL-138 (**B**) on endothelium-dependent vasodilation . ‘Control’ indicates measurement without inhibitor pre-incubation, ‘l-NAME’ indicates measurements in the presence of l-NAME, an inhibitor of endothelial nitric oxide synthase, and ‘L-NAME/apamin/TRAM-34’ indicates measurements in the presence of l-NAME and inhibitors of small and intermediate calcium-activated potassium channels (apamin, TRAM-34). Protein expression of cyclin-dependent kinase inhibitor 2A isoform p16INK4a (**P16**) and cyclin-dependent kinase inhibitor 1 (**P21**) in abdominal aorta (**C-D**), endothelium-independent vasodilation of thoracic aorta (**E**) and corresponding blot pictures for C-D (**F**). Data are shown as means ± SEM (**A, B, E**) and means ± SD (**C, D**). Significant effect of genotype: * or SUL-138: # (GLM with repeated measures (**A-B, E**) Kruskal–Wallis test (**C**) and one-way ANOVA (**D**) with *P*<0.05). Sample size for A and E: WT vehicle treated *N*=11–12, WT SUL-138 treated *N*=5–6; EC-KO vehicle treated *N*=8–10; EC-KO SUL-138 treated *N*=5–6; sample size for B: *N*=5 for all groups. mRNA expression of mitochondrial health markers in doxorubicin-stimulated HUVECs in the presence or absence of SUL-138 (G). Fis1, mitochondrial fission 1; Mfn1, mitofusin 1; Mfn2, mitofusin 2; Nrf1, nuclear respiratory factor 1; Opa1, optic atrophy 1; Tfam, mitochondrial transcription factor A. Data are shown as geometric means ± geometric SD. Significant effect of doxorubicin stimulation: * or SUL-138: # (two-way ANOVA).

In contrast with the chronic treatment, acute SUL-138 incubation did not affect vasodilation in either WT or EC-KO mice (Figure 2A). Overnight incubation with SUL-138 showed a similar trend as chronic treatment, including EDH modulation, albeit without reaching statistical significance (*P*=0.14) (Figure 2B). We confirmed cyclin-dependent kinase inhibitor 1 (P21) protein up-regulation [13], a marker for cell stress in senescence and aging, in EC-KO vehicle-treated aorta, which was significantly decreased by chronic SUL-138 treatment (Figure 2C and F). Similarly, elevated protein abundance of the senescence marker cyclin-dependent kinase inhibitor 2A isoform p16INK4a (P16) was observed in the aorta of EC-KO vehicle-treated mice, which was normalized by SUL-138 (Figure 2D and F). However, it needs to be noted that GAPDH expression varied between the groups. A similar variation was observed when correcting for total protein abundance using Ponceau protein staining (data not shown), suggesting variation in the input protein level, which is not uncommon with small aortic samples since they are difficult to process because of their fibrous structure and rigidness. Endothelium-independent vasodilation was similar in WT and EC-KO vehicle-treated mice and not altered by chronic SUL-138 treatment (Figure 2E). There were also no differences in blood pressure between the groups (Supplementary Table S3).

### Doxorubicin-stimulated human umbilical cord endothelial cells

To further obtain mechanistic insights of the action of SUL-138 on vascular aging, we stimulated HUVECs with doxorubicin to induce mitochondrial dysfunction and senescence, which are hallmarks of aging [12,35,37] in the presence or absence of SUL-138. The mRNA expression of mitochondrial health markers related to mitochondrial fusion and fission (*Mfn1, Mfn2, Opa1,* and *Fis1*) was significantly increased in doxorubicin-stimulated HUVECs compared with the control group. Moreover, the treatment with SUL-138 significantly reduced the mRNA expression of these markers compared with doxorubicin-treated cells (Figure 2G). Mitochondrial biogenesis markers *Tfam* and *Nrf1* were decreased after doxorubicin stimulation, which was unaffected by the treatment with SUL-138 (Figure 2G).

### Kidney function

EC-KO mice were previously shown to display increased microvascular leakage and senescence in the kidney [13]. We investigated if these renal changes were associated with functional and molecular glomerular and tubular damage markers (Table 1). Plasma creatinine and albumin levels were similar in all groups. Urinary creatinine excretion was higher in EC-KO vehicle-treated mice compared with WT vehicle-treated mice, but not affected by SUL-138 in EC-KO mice. Moreover, creatinine clearance was not statistically significant different between the groups. Furthermore, EC-KO vehicle-treated mice displayed albuminuria, which was not significantly lowered by the treatment with SUL-138.

**Table 1:** Kidney parameters measured in 24 h urine or blood plasma of vehicle or SUL-138 treated endothelial cell-specific Ercc1 KO mice (EC-KO) and wildtype (WT) mice. Values are expressed as means ± SD. Results are evaluated with two-way ANOVA with Bonferroni post hoc correction. *: significant effect of genotype or #: of SUL-138 with *P*<0.05

Parameter	WT vehicle treated (*N*=12)	WT SUL-138 treated (*N*=7)	EC-KO vehicle treated (*N*=12)	EC-KO SUL-138 treated (*N*=8)
Plasma creatinine, ng/ml	87.9 ± 20.2	92.7 ± 32.04	85.5 ± 14.1	97.1 ± 27.7
Plasma albumin, mg/ml	18.5 ± 3.0	19.0 ± 1.6	19.2 ± 4.2	19.2 ± 1.3
Plasma copeptin, ng/ml	10.0 ± 2.2	10.2 ± 1.7	10.7 ± 10.7	8.4 ± 2.2
Plasma sodium, mmol/l	148.5 ± 5.3	148.0 ± 2.7	149.7 ± 3.9	150.0 ± 3.2
Water intake, ml/day	3.1 ± 1.5	2.3 ± 0.5	5.2 ± 2.2*	3.1 ± 1.5 **^#^**
Food intake, mg/day	2.7 ± 1.3	2.2 ± 0.9	3.0 ± 0.9	2.2 ± 1.2
Urine volume, ml/day	0.5 ± 0.3	0.7 ± 0.1	2.2 ± 1.5*	0.8 ± 0.4**^#^**
Urine creatinine, mg/day	0.5 ± 0.2	0.9 ± 0.08**^#^**	0.8 ± 0.3*	0.8 ± 0.2
24 h creatinine clearance, ml/ h	2.6 ± 1.6	4.0 ± 1.1	3.6 ± 2.3	3.9 ± 2.0
Urine albumin/ creatinine [mg/g/day]	16.0 ± 6.4	9.6 ± 3.7	70.4 ± 45.2*	58.0 ± 43.0*
Urine sodium excretion, µmol/day	134.7 ± 33.9	155.4 ± 71.2	230.8 ± 89.7*	105.9 ± 31.9**^#^**
Urine potassium excretion, µmol/day	258.8 ± 101.9	216.2 ± 100.7	388.6 ± 114.0*	140.9 ± 42.5**^#^**
Urine glucose excretion, µmol/day	2.3 ± 0.8	2.2 ± 0.8	2.8 ± 0.9	1.3 ± 0.6**^#^**

Moreover, EC-KO vehicle-treated mice showed increased water intake and 24 h urine volume. Chronic SUL-138 treatment significantly reduced 24 h water intake and urine volume in EC-KO mice (Table 1). The hormones arginine vasopressin (AVP) and aldosterone regulate kidney water and sodium handling [38,39]. We previously showed that there were no changes in plasma aldosterone between EC-KO and WT (data not shown) and therefore now focused on AVP. Aging rodents demonstrate a reduced urinary concentrating ability, and this involves an altered responsiveness to AVP and decreased aquaporin 2 protein expression [40,41]. However, in our study, the levels of plasma sodium and plasma copeptin, a stable marker protein that is co-secreted with AVP in an equimolar manner, were not different between the groups (Table 1). Moreover, neither aquaporin 2 protein expression nor type 2 vasopressin receptor mRNA expression in the kidney was different between the groups (Supplementary Figure S1B-C).

Sodium reabsorption decreases during aging, and this has been linked to lower sodium transporter protein abundances [42]. We therefore measured urinary sodium and potassium excretion, which was higher in EC-KO vehicle-treated compared with WT vehicle-treated mice and significantly recovered after chronic SUL-138 treatment (Table 1). Furthermore, food intake was also not significantly different between the groups (Table 1).

**Figure 3:**
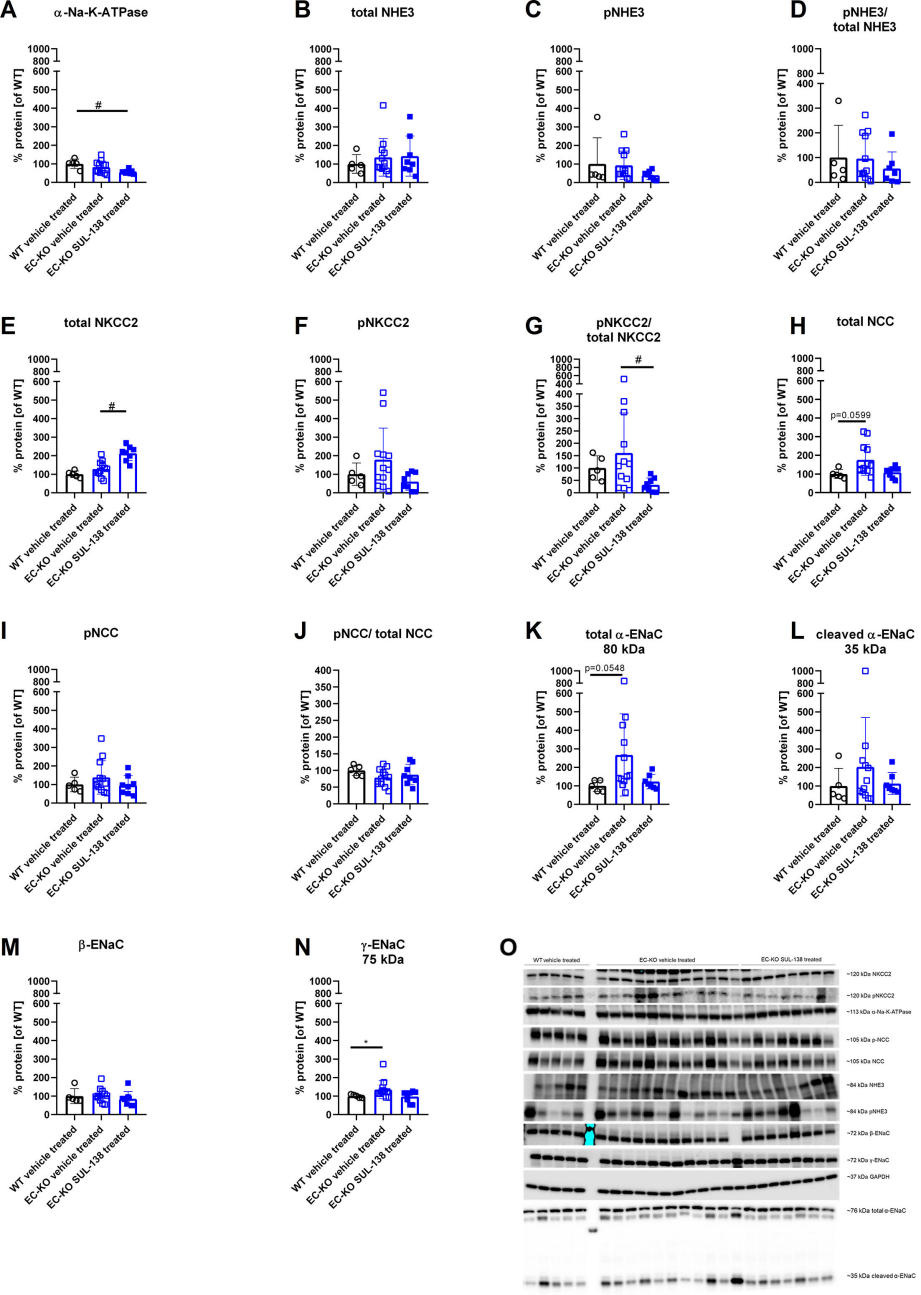
A: Protein expression of sodium transporters in kidneys from endothelial cell-specific Ercc1 KO mice (EC-KO) and wildtype (WT) mice. Abbreviations for proteins: Na-K-ATPase, sodium-potassium ATPase; NHE3, sodium-hydrogen-exchanger isoform 3; NKCC2, bumetanide-sensitive Na-K-2Cl cotransporter; NCC, thiazide-sensitive sodium chloride cotransporter; ENaC, epithelial sodium channel. The p in front of the abbreviation indicates phosphorylated protein, Greek letters indicate subunits of the proteins. Data are shown as means ± SD. *: significant effect of genotype or SUL-138: # (Kruskal–Wallis test (**B-G, I, K-N**) or one-way ANOVA with post-hoc test (**A, H, J**) with *P*<0.05) and O: corresponding Western blot pictures.

To investigate the mechanism underlying increased urinary sodium excretion in EC-KO mice, the protein abundances of kidney sodium transporters were analyzed (Figure 3).

The protein abundance of the catalytically active α-subunit of the sodium-potassium ATPase (α-Na-K-ATPase) was not different between EC-KO and WT vehicle-treated mice, but was significantly decreased after treating EC-KO mice with SUL-138 (Figure 3A).

There was no difference between the groups in protein abundance of total sodium-hydrogen-exchanger isoform 3, NHE3, located in the proximal tubule [43]. Moreover, neither the phosphorylated NHE3 protein abundance nor the ratio of phosphorylated/total protein was altered (Figure 3C–D). There were negligible glucose levels present in the urine of mice from all groups (Table 1).

The total protein abundance of the bumetanide-sensitive Na-K-2Cl cotransporter (NKCC2) in the thick ascending limb [43] was not different between WT and EC-KO vehicle-treated mice. However, the treatment with SUL-138 significantly increased total NKCC2 in EC-KO vehicle-treated mice (Figure 3E). Phosphorylated NKCC2 protein abundance was not different between the groups (Figure 3F). The ratio of phosphorylated/total NKCC2 was not different between EC-KO and WT vehicle-treated mice, but the treatment with SUL-138 decreased this ratio in EC-KO mice (Figure 3G).

In the distal convoluted tubule, sodium is reabsorbed through the thiazide-sensitive sodium chloride cotransporter (NCC) [43]. Total NCC protein abundance was higher in EC-KO vehicle-treated kidneys, but this difference did not reach statistical significance (*P*=0.06). SUL-138 lowered the protein abundance towards the level of WT vehicle-treated mice (Figure 3 A6). There were no significant differences in protein abundance of phosphorylated (active) NCC or the ratio of phosphorylated/total protein between the groups (Figure 3I–J).

In the collecting duct, the epithelial sodium channel (ENaC) is responsible for controlling the fine-tuning of sodium reabsorption [43]. The protein abundances of α-ENaC and γ-ENaC were significantly higher in EC-KO vehicle-treated mice and decreased after the treatment with SUL-138 (Figure 3K and N). A similar trend was observed for cleaved α-ENaC expression, which did not reach statistical significance (Figure 3L). β-ENaC protein abundance was not different between the groups (Figure 3M).

### Inflammation and cell stress response markers

**Figure 4:**
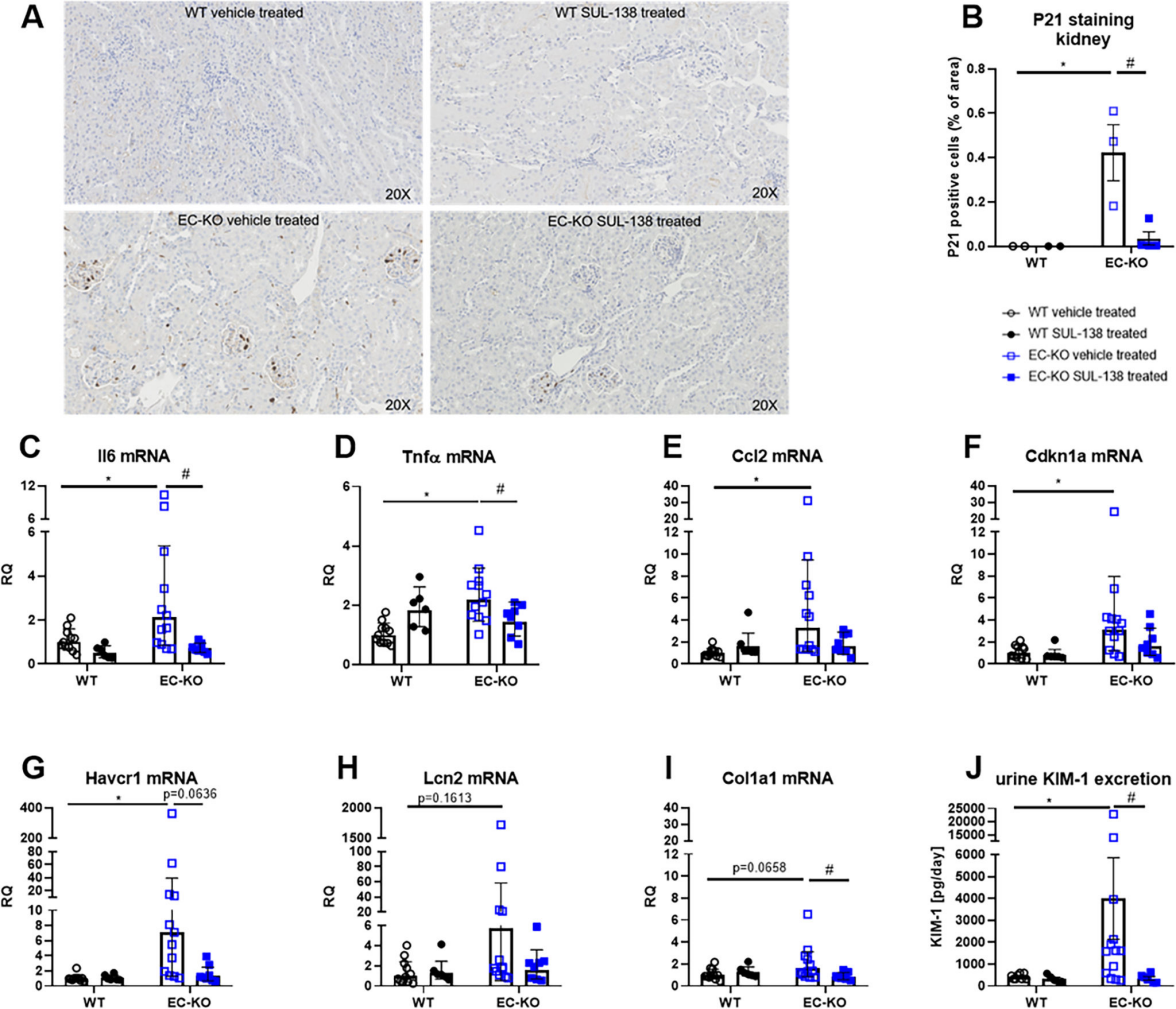
Senescence and cell stress marker expression in kidneys of endothelial cell-specific Ercc1 KO mice (EC-KO) and wildtype (WT) mice. P21 immunohistochemistry (**A**) and corresponding quantification of P21-positive cells (**B**), mRNA expression of inflammatory and kidney injury markers Interleukin 6 (Il6), tumor necrose factor alpha (Tnfα), CC motif chemokine ligand 2 (Ccl2), Cyclin-dependent kinase inhibitor 1A (Cdkn1a), hepatitis A virus cellular receptor 1 (Havcr1, Kim-1), lipocalin 2 (Lcn2, Ngal), collagen type I alpha 1 (Col1a1) (**C-I**), and kidney injury marker 1 (KIM-1) excretion (**J**). Data are shown as means ± SD (**A, J**) and geometric mean ± geometric SD (**C-I**). Significant effect of genotype: * or SUL-138: # (two-way ANOVA (**A, C-D, F, I-J**), and Kruskal–Wallis ranking test (**E, G, H**) with *P*<0.05).

DNA damage leads to a response characterized by inflammation, cell stress, and senescence [12]. Immunohistochemical staining for senescence marker P21 showed increased numbers of P21-positive cells in EC-KO vehicle-treated kidneys in glomeruli and blood vessels compared with WT vehicle-treated mice (Figure 4A–B). After the treatment with SUL-138, the number of P21-positive cells significantly decreased, and there were no P21-positive cells in kidneys of WT SUL-138-treated mice (Figure 4B). mRNA expression of inflammatory markers *Il6, Tnfα, and Ccl2* was increased in the kidney of EC-KO vehicle-treated mice, and expression of *Il6* and *Tnfα* was significantly reduced after the treatment with SUL-138 (Figure 4C–D). This was accompanied by augmented mRNA levels of *Cdkn1a* and kidney injury marker *Havcr1* in the kidneys of EC-KO vehicle-treated mice, which showed a trend of down-regulation after treatment with SUL-138 without reaching statistical significance (Figure 4F–G). mRNA expression of kidney injury marker *Lcn2* and fibrosis marker *Col1a1* showed a trend of being increased in EC-KO vehicle-treated mice without reaching statistical significance. However, *Col1a1* expression was significantly decreased in SUL-138 treated EC-KO mice compared with vehicle-treated EC-KO mice (Figure 4H–I). Moreover, urinary excretion of kidney injury marker KIM-1 was significantly increased in EC-KO vehicle-treated mice and normalized by treatment with SUL-138 (Figure 4J).

**Figure 5:**
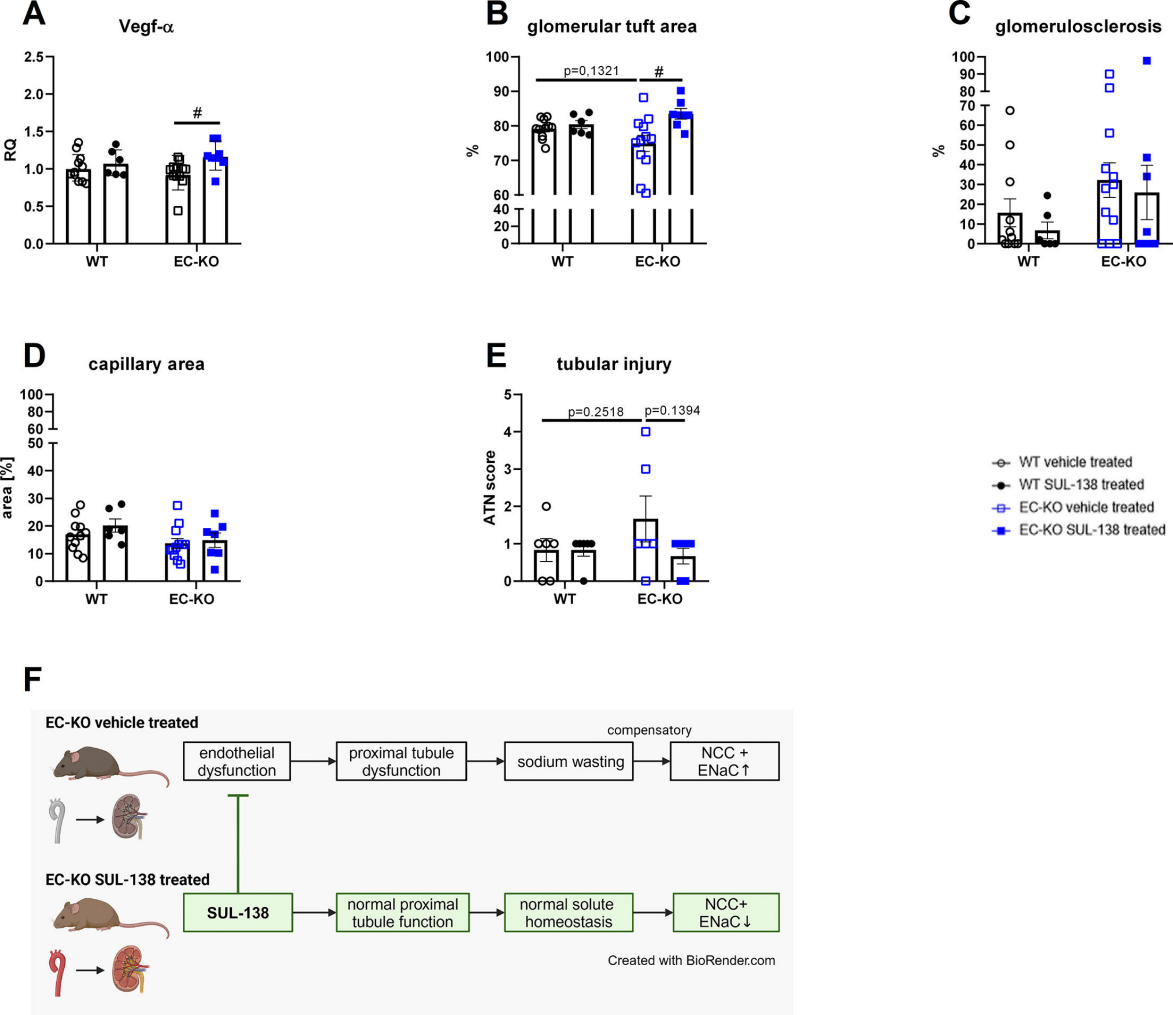
Histological assessment of the kidney from endothelial cell-specific Ercc1 KO mice (EC-KO) and wildtype (WT) mice. Vascular endothelial growth factor alpha (Vegf-α) mRNA expression in kidneys (**A**), Glomerular and tubular injury assessment in kidneys of EC-KO and WT mice evaluated with periodic acid–Schiff’s staining (**B-E**). Proposed mechanism of salt-wasting phenotype in EC-KO vehicle-treated mice and possible modulation by SUL-138 (**F**), created in BioRender.com. Data are shown as geometric mean ± SD (**A**) and means ± SD (**B-E**). #: significant effect of SUL-138 (two-way ANOVA with *P*<0.05). Tubular injury score was evaluated with a Mann–Whitney test with *P*<0.05.

Age-related EC dysfunction and decreased NO bioavailability contribute to impaired angiogenesis, which in turn leads to decreased blood supply and tissue injury [44]. We therefore measured mRNA expression of the angiogenesis factor *Vegf-α* in the kidney, which was not different between vehicle-treated WT and EC-KO, but significantly increased in SUL-138-treated EC-KO mice (Figure 5A).

Assessment of the PAS staining suggests no differences in glomerular tuft area, a proxy for renal microvascular function, between EC-KO and WT vehicle-treated mice. However, SUL-138 treatment significantly elevated glomerular tuft area (Figure 5B). Glomerulosclerosis and capillary area were not different between the groups (Figure 5C–D). Moreover, the tubular injury score was also not different between the groups (Figure 5E). Representative pictures of the PAS staining can be found in the supplemental material (Supplementary Figure S2).

## Discussion

In this study, we investigated the effect of chronic treatment with SUL-138, a mitochondrial protective compound, on vascular and kidney function in EC-KO mice. The treatment with SUL-138 preserved endothelial-dependent relaxation function by boosting the EDH contribution. In addition, the increase in DNA damage response-related cell stress and senescence markers P16 and P21 was inhibited by SUL-138. Furthermore, in doxorubicin-stimulated HUVECs, the treatment with SUL-138 decreased elevated mitochondrial fusion and fission genes. Accelerated EC aging in EC-KO mice was accompanied by proximal tubule dysfunction, resulting in KIM-1 up-regulation and sodium wasting. Rescue of vascular function by SUL-138 partly restored kidney function in EC-KO mice, especially where it concerns tubular function variables. The SUL-138-induced increase in glomerular tuft area, together with the augmented EDH effect in aorta, indicates that preservation of vascular function could explain the observed protective effect on kidney. Furthermore, the preserving effect of SUL-138 included attenuation of the DNA damage response and modulation of mitochondrial health-related genes.

The class of SUL compounds limits mitochondrial superoxide production and preserves mitochondrial membrane potential, mitochondrial complex IV function, and ATP production [26,28,29]. These protective effects were shown in a pre-clinical model of diabetes [29], a condition that is epidemiologically associated with aging and mimics vascular aging features [45]. One of these diabetes features is the loss of vascular NO-mediated signaling, against which SUL compounds protected by decreasing superoxide production [29]. In the present and a recent study [46], we have explored the vascular protective effects of SUL-138 in pre-clinical mouse models where accelerated aging was induced by ERCC1 DNA repair ablation in endothelial and vascular smooth muscle cells. Our results were distinct from those in the pre-clinical diabetes mouse model, in which mild aging features, such as partial loss of NO signaling, appeared [29]. In both accelerated vascular aging mouse models, vasodilator NO signaling was entirely lost, reflecting a severely progressed state of aging [13,46,47]. The loss of vasodilatory NO-signaling and partial compensation by EDH is a protective phenomenon that is observed in aging [16–18]. In both accelerated vascular aging models, the treatment with SUL-138 increased this compensation by EDH [46]. Thus, the treatment with SUL-138 appears to strengthen this protection mode in vascular aging. In parallel, mitochondrial protective characteristics relevant for aging are part of the pharmacological effects observed for SUL compounds. Firstly, decreased mitochondrial membrane potential, previously shown to be prevented by the treatment with SUL-138 [28], has been observed as a feature of mitochondrial dysfunction during aging [48,49]. Mitochondrial membrane potential maintenance is important for mitochondrial fusion, a process that endorses proper bioenergetic function and cellular resilience during aging [50–52]. The present study showed that doxorubicin exposure, a method to induce cellular aging, increased mitochondrial fusion markers in HUVECs, which were reduced by SUL-138. These results suggest that doxorubicin disturbs mitochondrial function by modulating mitochondrial fusion, and mitigation thereof by SUL-138 fits in its profile as a protective agent in aging as well as a compound that preserves mitochondrial membrane potential. Our results suggest that the improved vascular function through increased EDH mirrors an effect on aging by SUL compounds that derives from broad mitochondrial protection. It cannot be entirely excluded that SUL-138 pharmacologically targets the EDH pathway more directly. Changes in mitochondrial calcium signaling in microvascular endothelium leading to increased EDH have been postulated to support the adaptation during vascular aging [20]. The elevated EDH was linked to IK_Ca_- and SK_Ca_-channel activation [20], which are the same channels involved in mediating EDH in EC-KO mice. However, an immediate effect through this pathway does not seem to be a likely explanation, as SUL-138 did not have an acute vasodilatory effect. In conclusion, SUL-138 shows promising anti-aging effects in the vasculature by increasing EDH signaling, decreasing the DNA damage response, and modulating mitochondrial fusion. The hydrochloride salt of SUL-138 is currently under phase I clinical assessment in healthy volunteers and is to our knowledge the first clinically relevant compound with this beneficial effect on EDH.

EC-KO vehicle-treated mice displayed tubule injury marked by increased KIM-1, senescence, inflammation, and sodium wasting. This corroborates the findings of Cohen et al., who suggested that senescent ECs drive age-related kidney dysfunction [53]. *Ercc1^Δ/^* ^−^ mice, a progeria model with a truncated version of ERCC1 in all cells, display tubular degeneration and necrosis [32]. In *Ercc1^Δ/^* ^−^ mice, tubular cells are likely directly affected by EC-KO, explaining their kidney phenotype. The tubular injury in EC-KO vehicle-treated mice must represent a secondary effect of endothelial dysfunction, likely explaining the milder changes compared with *Ercc1^Δ/^* ^−^ mice. This hypothesis is supported by the lack of P21-positive tubular epithelial cells in EC-KO mice.

In contrast with *Ercc1^Δ/^* ^−^ mice, EC-KO vehicle-treated mice additionally display impaired sodium handling with sodium loss likely to result from changes in local renal sodium handling. Indeed, we found no evidence of changes in AVP and aldosterone signaling. Rather, increased KIM-1 levels in EC-KO vehicle-treated mice point to proximal tubule injury [54], which may explain the increased natriuresis and kaliuresis. Around 60–70% of total sodium reabsorption occurs along the proximal tubule, where NHE3 is predominantly responsible for the transport of sodium [43]. Loss of reabsorption in the proximal tubule is known to be compensated for by the up-regulation of α- and γ-ENaC [55]. In EC-KO mice, we indeed observe this compensatory response. The loss of reabsorption in the proximal tubule is not dependent on NHE3 protein levels in EC-KO mice, as shown by our Western blot. A remaining possibility is disturbed bioenergetic homeostasis in the proximal tubule cells. The proximal tubules are highly metabolically active and contain a high mitochondrial density to produce the required ATP for the solute transport that is driven by Na^+^/K-ATPase [56]. Mitochondrial dysfunction, including reduced mitochondrial ATP production, results in decreased solute transport and is a key mechanism in the pathophysiology of tubulopathies [56]. It is generally known that impaired renal vascular function leads to rapid decline of mitochondrial ATP production [57]. Increased microvascular leakage in the kidneys of EC-KO mice suggests impaired permeability of peritubular capillaries [13], leading to impaired renal vascular function. Furthermore, we previously showed endothelial dysfunction in coronary and iliac artery [13] as well as brain microvascular lesions [58]. Altogether, this suggests a parallel development of vascular dysfunction features in vascular beds of different embryological origin leading to impaired renal perfusion, which could result in declined mitochondrial ATP production in EC-KO mice. The increase in glomerular tuft area that is associated with the beneficial effects of SUL-138 points toward a contribution of increased renal perfusion to the renal protective effect of the treatment. The proposed mechanism is summarized in Figure 5F.

The current results showing an improvement of EDH are in line with two earlier studies. Firstly, the partial loss of EDH in coronary arteries from a diabetic mouse model was prevented by the mitochondrial reactive oxygen species scavenger Mito-Tempo [59]. Secondly, we recently showed that the water-soluble formulation of SUL-138, its hydrochloride salt (which was not yet available at the start of the present studies), corrected the loss of vasodilation due to DNA-damage response-related, accelerated aging of vascular smooth muscle cells in *Ercc1* mutant mice [46]. Selective vascular smooth muscle aging in these mice was accompanied by a mild increase in damage and inflammation markers in the kidney, which were also prevented by SUL-138 treatment. EC-KO mice have a far more dramatic renal phenotype compared with vascular smooth muscle cell aging mice, allowing us to more comprehensively study protective effects of preserving mitochondrial function. In the kidneys of EC-KO mice, the number of P21-positive cells in EC of the small arteries was increased in EC-KO vehicle-treated mice, similar to increased numbers of P21-positive cells in the aorta of EC-KO mice [13]. SUL-138 treatment decreased P21 in both kidney and aorta, indicating a similar effect of SUL-138 on EC in both vascular beds. Besides direct vascular effects of SUL-138, the treatment with SUL-138 also prevented sodium wasting in EC-KO mice and normalized the protein abundances of sodium transporters and levels of KIM-1 in EC-KO mice. It did not affect albuminuria, a phenomenon that either depends on glomerular leakage or disruption of tubular reabsorption [60]. The fact that sodium reabsorption is rescued and not albuminuria suggests that the rescue involves restoration of metabolically demanding active transport mechanisms in the proximal tubule. Therefore, we cannot exclude that part of the SUL-138 effect on tubular sodium reabsorption is due to modulation of mitochondrial function in tubular cells and does not solely rely on effects on endothelial cell function. To our knowledge, there is no model in which *in situ* effects of SUL-138 on mitochondrial function in aged vascular cells vs. proximal tubular cells of the kidney can be disentangled.

In summary, SUL-138 treatment rescues vascular function, and this is a plausible mechanism for prevention of the development of secondary tubular defects in the kidney. Targeting vascular aging to prevent age-related kidney injury might therefore be a promising pharmacological strategy (Figure 5F).

**Clinical perspectives**

- In this study, the role of mitochondrial dysfunction in DNA damage response-related endothelial aging and the impact on vascular and kidney function were investigated. This was done by studying the treatment effect of SUL-138, a novel drug that preserves mitochondrial complex IV function, mitochondrial membrane potential, and ATP production.
- We showed that impaired endothelium-dependent vasodilation in endothelial-cell-specific Ercc1 KO mice was fully restored after the treatment with SUL-138, which was driven by increased endothelium-derived hyperpolarization (EDH). This rescue of endothelial function was associated with preventing the development of renal tubular injury, inflammation, and sodium wasting.
- Vascular and kidney function decline with age independently from conventional risk factors such as diabetes, smoking, and hypertension [61]. The renal function decline could be a secondary defect resulting from non-obstructive vascular aging. Our measurements in the endothelium-selective accelerated aging mouse model support this thesis. Alleviation of mitochondrial dysfunction, an antagonistic hallmark of the biology of aging, with SUL-138 exerted therapeutic effects in this paradigm of pathogenesis. SUL-138 is currently being tested in a phase I clinical trial (NCT06277492) and might therefore be a readily translatable intervention drug in this context. In addition, we show that augmentation of EDH has therapeutic effects in vascular aging and related kidney injury. To our knowledge, this is the first paper showing such beneficial potential for EDH.

## Supplementary material

Online supplementary material 1

## Data Availability

The authors declare that all the data supporting the finding of this study are available within the paper or Supplemental Data.

## Open Access

This article has been published open access under our Subscribe to Open programme, made possible through the support of our subscribing institutions, learn more here: https://portlandpress.com/pages/open_access_options_and_prices#conditional

## Abbreviations

AVP: arginine vasopressin
EC: Endothelial cells
EC-KO: endothelial-cell specific Ercc1 KO
EDH: endothelium-derived hyperpolarization
ENaC: epithelial sodium channel
GLM: general linear model
HUVECs: human umbilical cord endothelial cells
IK_Ca_: Intermediate Ca^2+^-dependent potassium
KO: knock-out
K-channel: Potassium channel
NCC: thiazide-sensitive sodium chloride cotransporter
NKCC2: Na-K-2Cl cotransporter
NO: nitric oxide
PAS: periodic acid–Schiff’s
RT: room temperature
SD: standard deviation
SEM: standard error
SK_Ca_: small Ca^2+^-dependent potassium
WT: wildtype
