# Supplementary material for: SUL-138 mitigates accelerated endothelial aging and protects the kidney
Source: Clin Sci (Lond). 2025 Nov 6;139(21):1321–36. doi: 10.1042/CS20255735 (PMC12687458; doi:10.1042/CS20255735)
Supplement: Online supplementary material 1 [file cs-139-21-CS20255735-s001.docx]

**Supplemental material**


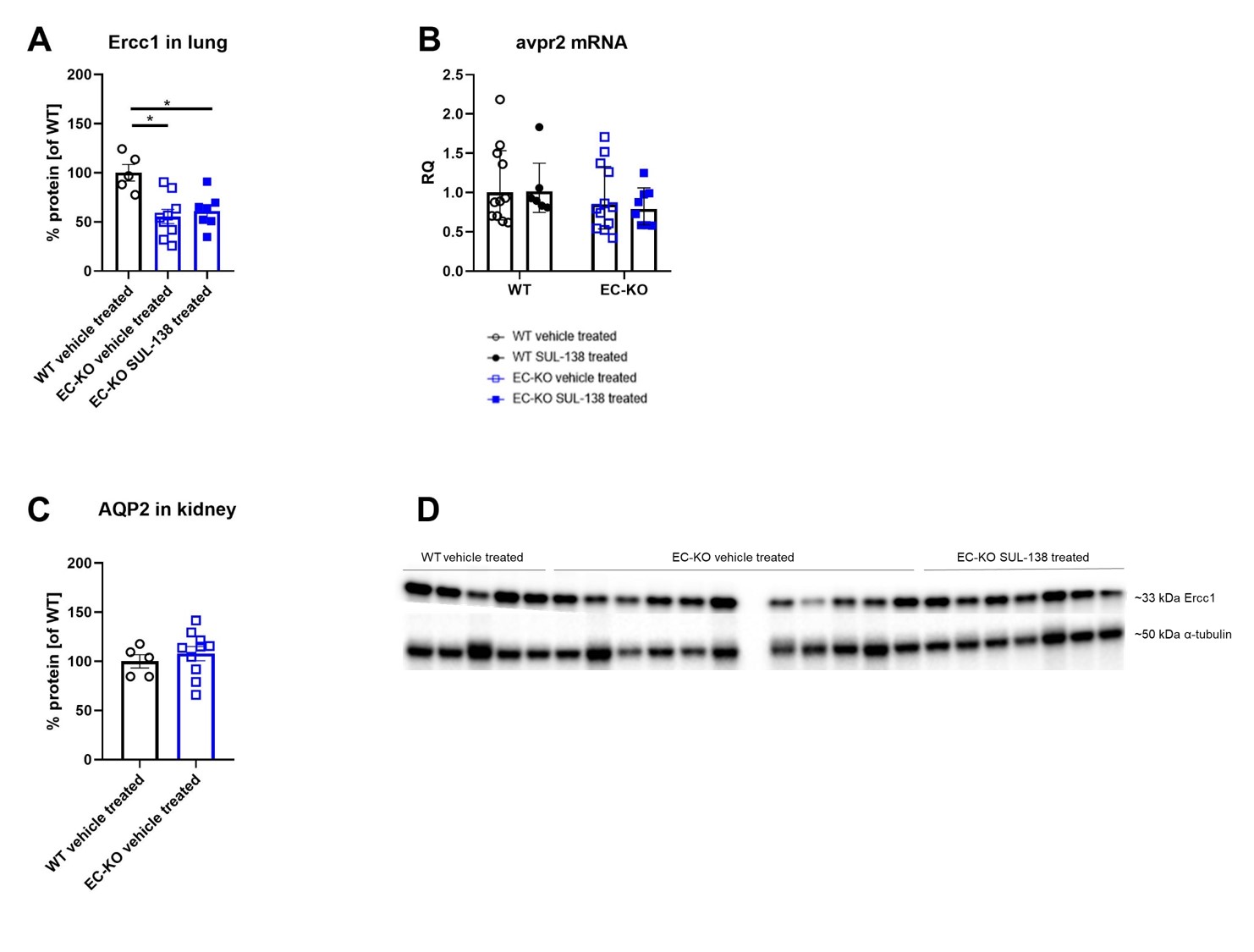


Supplemental Figure S1: ERCC1 protein expression in lung (A), vasopressin receptor 2 mRNA expression in kidney (B), and aquaporin 2 (AQP2) protein expression in kidney (C) from endothelial cell-specific *Ercc1* KO mice (EC-KO) and wild type (WT) mice. Associated blot picture can be found in D. *: significant effect of genotype (1-way ANOVA (A), 2-way ANOVA (B), unpaired t-test (C) with p<0.05).


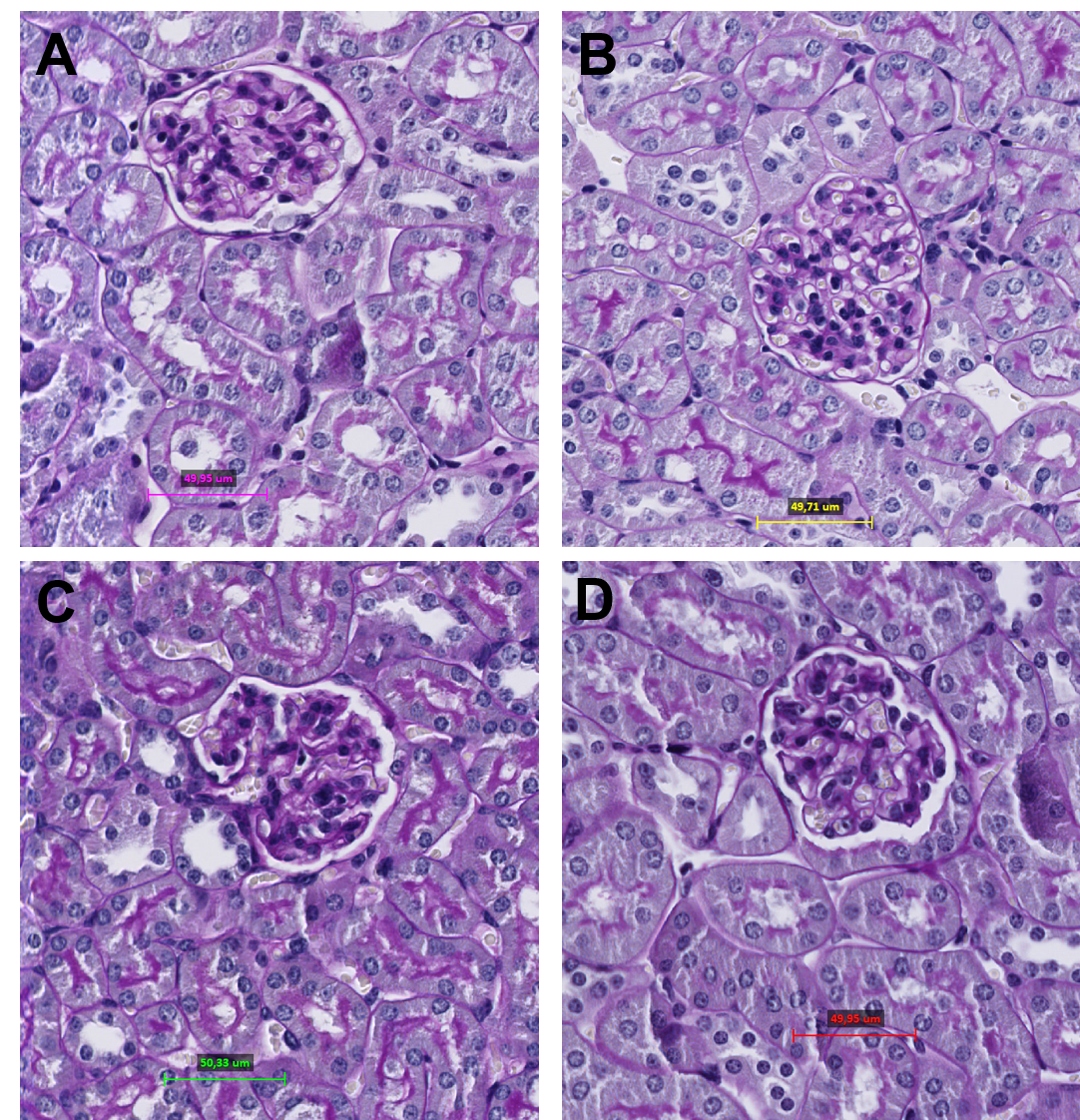


Supplemental Figure S2: representative picture for periodic acid – Schiff’s staining for wild type (WT) vehicle treated (A), WT SUL-138 treated (B), endothelial cell-specific *Ercc1* KO mice (EC-KO) vehicle treated (C), EC-KO SUL-138 treated mice.

Supplemental Table S1: Primary antibodies used for Western Blot.

| **Antibody target** | **concentration** | **supplier** |
| --- | --- | --- |
| Epithelial sodium channel, α-subunit, α-ENaC | 1:1000 | provided by Dr. Jan Loffing^a^ |
| Sodium-Potassium-ATPase, α–subunit, α-Na-K-ATPase | 1:1000 | ab7671, Abcam |
| Aquaporin 2, AQP2 | 1:500 | ab199975, Abcam |
| α-Tubulin | 1:1000 | sc5286, Santa-Cruz |
| Epithelial sodium channel, β -subunit, β-ENaC | 1:800 | SPC-404D, StressMarq |
| Epithelial sodium channel, γ -subunit, γ-ENaC | 1:10,000 | provided by Dr. Jan Loffing^a^ |
| Excision Repair Cross-Complementing Rodent Repair Deficiency, Complementation Group 1, ERCC1 | 1:1000 | ab129267, Abcam |
| Glyceraldehyde-3-phosphate dehydrogenase, GAPDH | 1:10,000 | 10494-1-AP, proteintech |
| Cyclin-dependent kinase inhibitor 2A isoform p16INK4a, P16 | 1:500 | 51-1325GR, BD Pharmingen |
| Cyclin-dependent kinase inhibitor 1A, P21 | 1:200 | sc-6246, Santa-Cruz |
| Thiazide-sensitive sodium chloride cotransporter, NCC | 1:1000 | SPC-402D, StressMarq |
| Sodium-hydrogen-exchanger isoform 3, NHE3 | 1:1000 | 27190-1-AP, proteintech |
| Phosphorylated sodium-hydrogen-exchanger isoform 3, pNHE3 | 1:200 | sc-53961, Santa-Cruz |
| Phosphorylated thiazide-sensitive sodium chloride cotransporter, pNCC (Thr 53) | 1:500 | provided by Dr. Rob Fenton^b^ |
| Bumetanide-sensitive Na-K-2Cl cotransporter, NKCC2 | 1:1000 | SPC-401D, StressMarq |
| Phosphorylated Bumetanide-sensitive Na-K-2Cl cotransporter, pNKCC2 | 1:500 | Provided by Dr. Kerim Mutig^c^ |

Supplemental Table S2: Primer sequences from primers used for qPCR.

| **Gene** | **Primer** | **Sequence** (5’ to 3’) |
| --- | --- | --- |
| **mouse** | | |
| *CC motif chemokine ligand 2 (Ccl2)* | *Ccl2* forward | AGCTGTAGTTTTTGTCACCAAGC |
|  | *Ccl2* reverse | TGTCTGGACCCATTCCTTCTTG |
| *Collagen type I alpha 1 (Col1a1)* | *Col1a1* forward | CCCTGGTCCCTCTGGAAATG |
|  | *Col1a1* reverse | GGACCTTTGCCCCCTTCTTT |
| *Interleukin 6 (Il6)* | *Il6* forward | TCCAGTTGCCTTCTTGGGAC |
|  | *Il6* reverse | GTGTAATTAAGCCTCCGACTTG |
| *hepatitis A virus cellular receptor 1 (Havcr1, Kim-1)* | *Havcr1* forward | CAAACCAGACTGGAATGGCA |
|  | *Havcr1* reverse | CTGGAGGGATTGCTTCAGTGT |
| *Lipocalin 2 (Lcn2, Ngal)* | *Lnc2* forward | GGAACGTTTCACCCGCTTTG |
|  | *Lnc2* reverse | CCACACTCACCACCCATTCA |
| *Cyclin-dependent kinase inhibitor 1A (Cdkn1a)* | *Cdkn1a* forward | CAGACCAGCCTGACAGATTTC |
|  | *Cdkn1a* reverse | GTTTTCTCTTGCAGAAGACCAATCT |
| *Tumor necrose factor alpha [Tnfα]* | *Tnf-alpha* forward | GTCCCCAAAGGGATGAGAAGTT |
|  | *Tnf-alpha* reverse | GCTACAGGCTTGTCACTCGAA |
| *Vascular endothelial growth factor alpha (Vegf-α)* | *Vegf-alpha* forward | AAAACACAGACTCGCGTTGC |
|  | *Vegf-alpha* reverse | CCTTGGCTTGTCACATCTGC |
| *Beta actin* | *Beta-actin* forward | CACTGTCGAGTCGCGTCC |
|  | *Beta-actin* reverse | TCATCCATGGCGAACTGGTG |
| *Glyceraldehyde-3-phosphate dehydrogenase (Gapdh)* | *Gapdh* forward | GTGCAGTGCCAGCCTCGT |
|  | *Gapdh* reverse | GAAGGGGTCGTTGATGGCAA |
| **human** | | |
| *Mitochondrial fission 1* | *Fis1 forward* | GGAACTACCGGCTCAAGGAAT |
|  | *Fis1 reverse* | TTGTTCTGGGGCTCTGTCTG |
| *Mitochondrial transcription factor A* | *Tfam forward* | CCAAAAAGACCTCGTTCAGCTT |
|  | *Tfam reverse* | CTTCAGCTTTTCCTGCGGTG |
| *Mitofusin 1* | *Mfn1 forward* | AGTTGGAGCGGAGACTTAGCA |
|  | *Mfn1 reverse* | TCGCCTTCTTAGCCAGCACA |
| *Mitofusin 2* | *Mfn2 forward* | GAAGTGGAGAGGCAGGTGTC |
|  | *Mfn2 reverse* | GTCCTTCCTCTATGTGGCGG |
| *Nuclear respiratory factor 1* | *Nrf1 forward* | CGCAGCCGCTCTGAGTAGAA |
|  | *Nrf1 reverse* | CGGTGTAAGTAGCCACATGGA |
| *Optic atrophy 1* | *Opa1 forward* | CCACTTCCTGGGTCATTCCT |
|  | *Opa1 reverse* | GCAGACCTCACAGGCCAC |
| *Beta actin* | *Beta-actin* forward | CTCCCTGGAGAAGAGCTACG |
|  | *Beta-actin* reverse | GAAGGAAGGCTGGAAGAGTG |
| *Hypoxanthin-Guanin-Phosphoribosyltransferase* | *Hprt1 forward* | TGACACTGGCAAAACAATGCA |
|  | *Hprt1 reverse* | GGTCCTTTTCACCAGCAAGCT |
| *Glyceraldehyde-3-phosphate dehydrogenase (Gapdh)* | *Gapdh* forward | TGCACCACCAACTGCTTAGC |
|  | *Gapdh* reverse | GGCATGGACTGTGGTCATGAG |

Supplemental Table S3: Blood pressure measured with the tail cuff method in endothelial cell-specific *Ercc1* KO mice (EC-KO) and wild type (WT) mice.

| **Parameter** | **WT vehicle treated** | **WT SUL-138 treated** | **EC-KO vehicle treated** | **EC-KO SUL-138 treated** |
| --- | --- | --- | --- | --- |
| Diastolic blood pressure [mmHg] | 96 ±4 | 97 ±4 | 100 ±3 | 93 ±5 |
| Systolic blood pressure [mmHg] | 127 ±4 | 128 ±4 | 133 ±3 | 124 ±5 |

**References**

^a^: Picard N, Trompf K, Yang CL, Miller RL, Carrel M, Loffing-Cueni D, Fenton RA, Ellison DH, Loffing J. Protein phosphatase 1 inhibitor-1 deficiency reduces phosphorylation of renal NaCl cotransporter and causes arterial hypotension. J Am Soc Nephrol. 2014; 25:511-522.

^b^: Pedersen NB, Hofmeister MV, Rosenbaek LL, Nielsen J, Fenton RA. Vasopressin induces phosphorylation of the thiazide-sensitive sodium chloride cotransporter in the distal convoluted tubule. Kidney Int. 2010;78:160-169.

^c^: Mutig, K., A. Paliege, T. Kahl, T. Jöns, W. Müller-Esterl and S. Bachmann (2007). "Vasopressin V2 receptor expression along rat, mouse, and human renal epithelia with focus on TAL." American Journal of Physiology-Renal Physiology 293(4): F1166-F1177.
